# The Potential of Steroid Profiling by Mass Spectrometry in the Management of Adrenocortical Carcinoma

**DOI:** 10.3390/biomedicines8090314

**Published:** 2020-08-28

**Authors:** Claudia Rossi, Ilaria Cicalini, Sara Verrocchio, Giulia Di Dalmazi, Luca Federici, Ines Bucci

**Affiliations:** 1 Center for Advanced Studies and Technology (CAST), University “G. d’Annunzio” of Chieti-Pescara, 66100 Chieti, Italy; ilaria.cicalini@unich.it (I.C.); sara.verrocchio@gmail.com (S.V.); giulia.didalmazi@unich.it (G.D.D.); lfederici@unich.it (L.F.); ines.bucci@unich.it (I.B.); 2 Department of Medicine and Aging Science, University “G. d’Annunzio” of Chieti-Pescara, 66100 Chieti, Italy; 3 Department of Medical, Oral and Biotechnological Sciences, University of Chieti “G. d’Annunzio”, Via dei Vestini 31, 66100 Chieti, Italy

**Keywords:** adrenocortical carcinoma, steroid profiling, mass spectrometry, metabolomics, LC-MS/MS, adrenocortical disorders

## Abstract

Radiological and endocrinological work up of adrenal neoplasms is aimed at distinguishing between frequent non-functioning adenomas and rare but very aggressive adrenocortical carcinoma (ACC). Relevant research has addressed the identification of molecular, genetic and hormonal markers that could have clinical significance for malignancy, as well as a prognostic value. Regarding endocrine aspects, attention has been paid to the pattern of steroid secretion that can be affected by altered steroidogenic pathway in ACC. The advent of mass spectrometry techniques has overcome many limitations usually associated with immunoassays, allowing the determination of both common and rarely measured steroids in a single analysis with high specificity and sensitivity. Indeed, mass spectrometry strategies may be able to identify an individualized steroid profile of ACC, allowing a rapid diagnosis and a specific follow-up. In this review, insights, strengths and limitations of mass spectrometry-based approaches in steroid profiling, as well as of immunoassay in steroid measurements, will be specifically discussed. Moreover, the latest findings on steroid profiling by mass spectrometry-based techniques, the most promising analytical tool, will be summarized to evaluate if steroid profiling might be the clue for solving the clinical dilemma in differentiating ACC from non-functioning adrenocortical adenomas (ACA).

## 1. Introduction

Adrenocortical tumors (ACTs) are very common and frequently discovered incidentally on imaging performed for unrelated diseases. Most ACTs are small, benign, non-functioning adenomas. ACC, in contrast, is very rare and highly aggressive [1]. ACC is an orphan malignancy that has received increasing scientific attention in the last decades [2]. Relevant research on molecular pathogenesis, hormonal, genetic and epigenetic studies have been directed to clarify adrenal tumorigenesis and to identify markers of malignancy and/or prognostic significance, as well as potential therapeutic targets. Despite this increased knowledge on ACC, there are still areas of uncertainty regarding diagnosis, therapy and follow-up [2]. To date, the evaluation of malignancy risk mainly relies on radiological examination, even if recent guidelines aim at reducing such radiological assays in the follow-up of the patient given that the psychological burden for patients seems to increase with repeated imaging assays [3,4]. Thus, one of the controversial aspects is whether sensitive plasma/urine steroid hormone profiling by mass spectrometry (MS) approaches may improve diagnostic accuracy at initial work up and during follow up of ACC [4]. The perspective of a MS-based steroid profiling for ACC diagnosis acquires more value when considering that a steroid hormones excess has been observed in the majority of ACC patients (specifically 60–70%), even if not clinically evident in many of them [5]. This excess of steroids may be explained by a relatively inefficient steroid production in ACC, resulting from enzyme deficiencies in steroidogenesis and leading to a buildup of steroid precursors [6,7]. Therefore, the present work is addressed to review the potential role of steroid profiling in different biological fluids by MS-based techniques in ACC. Hence, the latest findings in monitoring steroids by MS-based strategies will be discussed to evaluate if steroid profiling may open the way for solving the clinical dilemma in differentiating ACC from non-functioning ACA. Furthermore, insights, strengths and limitations of the most promising analytical tool will be specifically discussed and compared to traditional and widely used immunometric techniques for steroid assays.

## 2. The Clinical Challenge in the Diagnosis and Management of Adrenocortical Carcinoma

ACC is a very rare and highly aggressive malignancy [1] with a mean 5-year survival rate between 16% and 47%, dramatically dropping to 5–10% in metastatic disease [8]. The incidence is reported to be 0.7–2.0 cases per million per year [9]. A peak incidence between 40 and 50 years, and a female prevalence have been described [2]. A bimodal distribution is also observed, and ACC is relatively frequent in children representing 1.3% of all childhood cancers as opposed to 0.02% to 0.2% of adult [10]. ACC may be either sporadic or familiar. Childhood ACC is a core malignancy of Li Fraumeni syndrome (LFS). Other familiar forms are Beckwith-Wiedemann syndrome, Gardner syndrome, Multiple Endocrine Neoplasia type 1 (MEN1), MEN 2, McCune-Albright syndrome, and Carney complex [11,12]. Germline mutations of p53 are present in about 4% of patients with ACC and in 70% of patients with LFS [13]. Other genes involved in the pathogenesis of ACC include: CTNNB1 gene (β-catenin), PRKARIA, Ras genes, TP53, GNAS, and MEN1 [14]. The two most frequent alterations observed in ACC are the overexpression of IGF-2 and the constitutive activation of the Wnt/β-catenin pathway, although driving mutations remain incompletely understood. Clinical presentation of ACC is related to signs and symptoms of adrenocortical hormone excess in 40–60% of the patients; in 20–30% of cases ACC is diagnosed incidentally by imaging studies for unrelated medical issues; in almost one third of the cases patients complain for back pain, abdominal pain/discomfort due to tumor mass effect [2]. The majority of ACC are functioning. A total of 50–80% of hormonally active ACC produce cortisol causing classic, pronounced and rapidly progressive cushingoid features [1]. Hypokalemia can develop due to glucocorticoid-mediated mineralocorticoid receptor activation by the very high levels of cortisol. A total of 40–60% of hormone-secreting ACC produce adrenal androgen causing hirsutism, virilization, and menstrual irregularities in women, and rapid-onset male pattern baldness in men. Mixed androgen and cortisol secretion are frequently observed. Symptoms of estrogen hypersecretion (gynecomastia and testicular atrophy) are present in 5–10% of male patients and are highly suspicious for ACC. Excess of estrogens is observed also in androgen secreting ACC due to their peripheral conversion [1]. Less frequently, ACC produce aldosterone causing hypertension and hypokalemia that, however, can be observed also when ACC produces mineralocorticoid precursors, such as 11-deoxycorticosterone [15].

## 3. Diagnostic Workup and Treatment

Imaging and hormonal studies are mandatory in patients with adrenal lesions and the diagnostic workup is aimed at distinguishing between frequent non-functioning ACA and rare but very aggressive ACC. A prompt diagnosis is necessary for an early therapeutic intervention to avoid tumor progression in case of ACC [4,6]. Imaging features and hormonal pattern have a pivotal role not only at initial diagnosis and staging but also during the follow up to monitor the response to treatment of patients with ACC.

### 3.1. Imaging

The detailed description of adrenal imaging is beyond the scope of this work and is well addressed elsewhere [8,16,17,18]. Briefly, key imaging features to differentiate benign and malignant adrenal lesions include size, vascularity, presence/absence of intracytoplasmic lipid, fat cells, hemorrhage, calcification, or necrosis, FDG avidity, and locoregional and distant disease. Unenhanced computed tomography (CT) is the first line imaging test for differentiating benign from malignant adrenal lesions. Although the risk of malignancy is higher with the increase of the diameter of adrenal mass, and most of ACCs are generally large tumors, measuring on average 10 to 13 cm, the size alone is not sufficient for the prediction of such risk [6] due to the overlap in size among ACCs, adenomas, metastases, and pheochromocytomas. Furthermore, features such as tumor heterogeneity, lobulated shape, irregular margins, and calcifications also lack in specificity. The measurement of the attenuation of adrenal lesions has a significant role and a threshold value of 10 Hounsfield unit (HU) is routinely used for the diagnosis of lipid-rich adenomas; lesions with an attenuation value greater than 10 HU on unenhanced CT are worth of additional studies. A study on a large series of ACC report that, although the vast majority of ACCs have unenhanced HU >21, a threshold of 13 should be used to avoid misdiagnosing an ACC as benign [19]. A slow contrast wash out on enhanced CT has been reported for ACC [20]. Enhanced CT is the technique of choice to assess organ invasion and metastatic spread, feasibility of radical surgery, and assessment of response to treatment. Magnetic resonance imaging (MRI) is, overall, as accurate as CT in the differentiation between benign and malignant adrenal masses [16]. The loss of signal intensity on out-of-phase images in relation to a reference organ reflects the presence of intra-cytoplasmatic fat, a feature typical of adenomas. Conversely, lipid-poor lesions, as metastases, pheochromocytomas, or ACC, do not show any change in signal intensity on out-of-phase images [8]. FDG-PET and/or PET/CT is widely used to characterize indeterminate adrenal lesions or lesions suspected of malignancy at first line imaging [21] and to assess recurrence during the follow up of ACC patients. Non-functional adrenal adenomas typically do not show increased FDG uptake and a certain form of functional adenoma (especially cortisol secreting) could present various FDG uptake. An adrenal to liver max standardized uptake value (SUV) ratio of between 1.45 and 1.8 has been suggested to separate between benign and malignant adrenal lesions. False negatives have been described due to small size of lesion (<1 cm), low FDG avidity of certain cancer types (such as renal cancer or neuroendocrine tumors), or necrosis within the tumor. In patients with ACC, 18F-FDG PET is recommended in staging disease and to evaluate patients for local recurrence and distant metastases [8]. The use of FDG PET/CT as a second-line test in the post-operative surveillance of ACC patients following CT finding of a potential recurrence may have a significant impact on patient management [22].

### 3.2. Endocrine Workup

A complete hormonal evaluation is mandatory in all patients with adrenal mass in order to distinguish functioning and non-functioning lesions, to characterize which hormones are secreted, to predict the risk of life-threatening post-operative adrenal insufficiency in patients with cortisol secreting tumors, to exclude catecholamine and avoiding life-threatening hypertensive crisis during surgery in case of pheochromocytomas and, mostly, to identify a hormonal tumor marker that may help predicting persistence or recurrence of disease during the follow-up [8].

The hormonal evaluation is necessary in both symptomatic and asymptomatic patients. Demonstration of autonomous, ACTH independent, cortisol secretion follows the recommendation for diagnosis of Cushing syndrome: 1-mg dexamethasone suppression test (1-mg DST), urinary-free cortisol (UFC), and late-night salivary cortisol (LNSC) [23,24]. The indications, evidence, strengths, and limitations of these tests are reviewed in the Endocrine Society Clinical Practice Guidelines [23].

Post DST cortisol levels between 1.9–5.0 µg/dL should be considered as evidence of possible autonomous cortisol secretion and cortisol levels >5.0 µg/dL should be taken as evidence of autonomous cortisol secretion. Additional biochemical tests to confirm cortisol secretory autonomy and assess the degree of cortisol secretion might be required [24].

Cortisol secretion is reported as a negative prognostic factor in ACC patients [25]. In a large series of patients with completely resected ACC, the prognostic significance of cortisol excess was high for both recurrence free survival and overall survival after adjustment for sex, age, tumor stage, and mitotane treatment [26].

Aldosterone-producing ACC is rare and is generally associated with hypertension and severe hypokalemia. Demonstration of aldosterone hypersecretion may require time due to the necessity of pharmacologic washout of interfering drugs [27] and thus can be skipped to avoid a delay of surgery [3,27]. Hormonal assessment must include sexual steroids (testosterone, estradiol). Estrogens hypersecretion has to be ascertained in males and postmenopausal females and is nearly pathognomonic of ACC. 17-Hydroxyprogesterone (17-OHP), androstenedione and Dehydroepiandrosterone Sulfate (DHEAS) must be also included in the hormonal assessment since they are often increased in ACC. Tests aimed at demonstrating catecholamine overproduction should be performed prior to surgery in all patients affected by adrenal masses [28].

It is worth mentioning that the diagnosis of steroid hormones secretion is critically dependent on the quality and performance of assays and on the technique employed (immunoassays, MS). By the use of sensitive methods, such as gas chromatography-MS, steroid hormones excess can be demonstrated in up to 95% of ACC cases, even if this excess is not always clinically evident [5]. A possible explanation may be a relatively inefficient steroid production in ACC, resulting from decreased expression of several steroidogenic enzymes leading to a buildup of steroid precursors [6,7]. Indeed, disorganized steroidogenesis has been histologically proved in ACC; lack of the coordinated expression of steroidogenic enzymes results in inefficient production of steroid hormones in relation to the large tumor volume causing mild clinical features [29].

In this context, the application of steroid profiling by MS-based approaches may reveal its potential in the diagnosis and management of ACC.

The role of different techniques in steroid determination in patients with adrenal lesions and ACC is discussed below.

### 3.3. Treatment

Currently, the only curative approach to ACC is complete tumor resection, while treatment strategies and follow-up of ACC depend strongly on the stage of the disease [8,16,17,18,30]. Briefly, for localized ACC (stages I, II, some III), radical surgery is aimed at complete tumor resection. For unresectable or metastatic ACC, all therapy must be considered palliative, even if in some cases surgery may be suggested if complete resection of all lesions seems feasible. Mitotane is the main drug for ACC treatment, both as adjuvant treatment after complete resection and in recurrent, inoperable and/or metastatic ACC [31], due to the capacity to block the steroidogenesis and to exert cytotoxic effect on adrenocortical cells. Adjuvant therapy with Mitotane is suggested in patients without macroscopic residual tumor after surgery but who have a high risk of recurrence and it can be also recommended for patients at low/moderate risk of recurrence. Mitotane should be started as soon as clinically possible in patients with advanced unresectable ACC [31]. Other therapeutic options are chemotherapy (etoposide/doxorubicin/cisplatin) and radiotherapy, while data emerging from molecular, case report, and phase 2 trials hopefully forecast next-generation therapies of ACC (targeted therapies and immunotherapy).

## 4. Steroid Measurement by Immunoassay

Immunoassays (IA) are bioanalytical methods in which the quantitation of an analyte, the antigen, depends on its recognition by a reagent antibody directed against it. IA over the past 50 years has been regarded as the technique of choice for steroid analysis and became an important tool in clinical endocrinology laboratories [32]. Immunological approaches for steroid quantifications comprise radioimmunoassays (RIA), enzyme-linked immunosorbent assays (ELISA), and fluoroimmunoassays (FIA) [33].

They offer several advantages, but also some limitations as summarized in Table 1.

### 4.1. Advantages of IA

Traditionally, immunoassay steroids quantification requires small quantities of various types of samples (serum, urine, saliva) and the sample preparation is a very simple step. Commercial kits, reagents, and automated low-cost instrumentation are easily available, making the analysis accessible even to small laboratories and unskilled technicians. In fact, contrary to analyses that require chromatographic separation, extraction and the fractionation phases are rarely used. Thus, IA are easy to set up even if only one analyte can be measured per immunoassay [32,34]. Results are reliable, if quality controls measures are in place and reference ranges of some steroids are fully accepted by the clinician community, considering that the advent of IA in endocrinology practice dates back to the 60s. This represents a huge advantage in clinical application and it is probably the reason why this approach, despite the numerous disadvantages associated with it, is still widely used.

### 4.2. Disadvantages of IA 

The disadvantages of IA are well known and include the potential lack of specificity, the risk of cross reactivity with structurally similar molecules and standardization issues between laboratories [35].

The potential lack of specificity is usually due to the antiserum-antibody bounding to the target molecule. In the case of steroids, the target molecule is very small when compared to the antibody binding site, and therefore steroids with a very similar chemical structure, although with a very different function, could bind the same antibody [35,36,37]. As a result, an overestimation of analyte concentrations could occur especially when the cross-reactive steroid concentrations are higher compared to the analyte of interest. This phenomenon may be less evident when the concentrations of the interfering molecules are lower compared to the analyte [35].

As regards to sensitivity, the quantification of the steroids in IA is limited to the linear portion of the standard curve, observing an increase in the standard error in the extreme portions of the quantification curve. Over time, however, many efforts have been made to improve this aspect, including the computer-based curve fitting tool [32].

### 4.3. Potential Pitfalls of Steroid Measurement by Immunoassay

The biological fluids most used for steroid analysis in IA are serum, urine, and saliva [34,38]. Most measured steroids using IA are cortisol, estradiol (E2), and testosterone. Current assays measure total serum bound and unbound cortisol and as a result are affected by changes in Cortisol Binding Globulin and albumin concentrations. Increased binding protein concentration, which occurs with estrogens excess (pregnancy, estrogens-containing oral contraceptives), can cause higher concentrations of cortisol while falsely low cortisol levels can be observed in acutely ill patients [39]. Interference by precursors makes cortisol assay unreliable in patients treated with steroidogenesis inhibitors such as metyrapone. In addition, free urinary cortisol determination can be affected by cross reactivity of metabolites resulting in overestimation of the hormone concentration. Salivary cortisol has been suggested as a convenient alternative to serum since it reflects cortisol’s circadian rhythm, and responds to changes in plasma cortisol concentration quickly and reliably [40]. Automated serum cortisol immunoassays have been successfully adapted to measure cortisol in saliva and late-night salivary cortisol is a recommended first-line screening test for Cushing’s syndrome. The specificity of salivary cortisol can be limited by other saliva components, such as the structurally related cortisol metabolite, cortisone, or the highly abundant salivary protein α-amylase [34]. Relevant to the focus of the present work is also the role of cortisol measurement during treatment with mitotane. Mitotane blocks steroidogenesis is at the level of 20, 22-desmolase, and 11β-hydroxylase, therefore inducing adrenal insufficiency and thus requiring hydrocortisone replacement therapy. Assessment of the adequacy of replacement therapy is hardly feasible on the basis of symptoms and quality of life in oncologic patients; plasma cortisol cannot be reliable since mitotane increases cortisol binding globulin levels and also urinary free cortisol may be falsely elevated due to the drug induced corticosteroid catabolism. In this setting, salivary cortisol assay and steroid profile by MS may be more effective in monitoring replacement therapy in these patients [41].

Testosterone and estradiol measurements by IA have been regarded as inaccurate in some clinical settings, claiming MS techniques as the method of choice. Furthermore, estradiol IA suffer from low accuracy, especially when detection of low levels is required in women, and also because male serum levels often fall below the sensitivity of the assay. Despite these limits, it is worth remembering that recent and well standardized IA can be considered suitable in clinical practice [42], since the measurement of “traditional” steroids, such as cortisol, estradiol, and testosterone, is considered a very accessible and economic technique, thanks to the large availability of ready-to-use kits.

Conversely, the development of the less frequently used niche tests for the quantification of different steroids in routine clinical practice may become costly and laborious. Among the steroids less commonly measured in IA there are: 11-deoxycorticosterone, aldosterone, 17-OHP, DHEA, and dihydrotestosterone (DHT), which are all readily measured using MS approaches [34].

Finally, quantifying steroids by IA is considered an easy and cost-effective technique, but it allows one to measure one analyte per assay, while adding an extra test may become extremely expensive. Moreover, multiplexing analysis is the main disadvantage of IA and, at the same time, the strength of MS-based analysis, as discussed below and summarized in Table 1.

## 5. Steroid Profiling by Mass Spectrometry-Based Approach

Laboratory testing play a pivotal role in clinical practice, especially for endocrinology, since steroid quantification is considered the most troublesome determination in clinical routine [43].

MS-based methods allow a high-resolution chromatographic separation, especially for steroids with similar chemical structures, and provide rapid and reproducible results with excellent purification.

**Figure 1:**
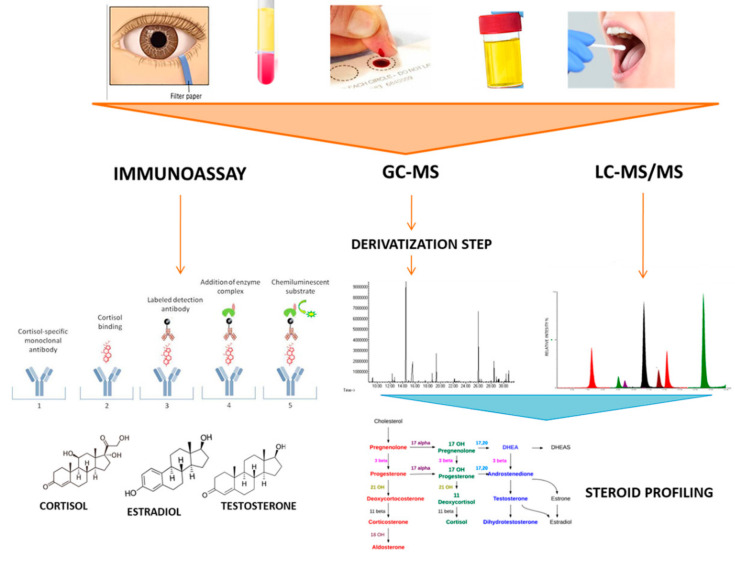
The Figure illustrates the main techniques for steroid determination in different biological fluids: Immunoassays, Gas Chromatography-Mass Spectrometry (GC-MS) and Liquid Chromatography-Tandem Mass Spectrometry (LC-MS/MS).

During the past 15 years, the advent of metabolomics has allowed the determination of steroid profiling by mass spectrometry-based approaches, overcoming many of the limitations associated to IA [44]. Obviously, the key strength of MS-based approach, that is, the possibility of multiplexing steroid tests rather than the measure of one steroid alone, is more relevant in complex adrenal diseases, as adrenal tumor and congenital adrenal hyperplasia, and is considered as a powerful diagnostic tool [45]. Gas chromatography coupled to mass spectrometry (GC-MS), with the use of isotopically labeled internal standards, has been long viewed as the “gold standard” method for multiple steroid determinations but, being time consuming, its use is not appropriate for routine clinical analysis [46]. In both the older GC-MS and the younger LC technique combined with tandem MS (LC-MS/MS), the clue for selectivity relies on chromatography [45]. More recently, the new imaging applications through the MALDI-MSI approach for the spatial distribution of endogenous and exogenous tissue analytes, are also used as a promising tool in clinical applications based on the analysis of the distribution of cortisol, Aldosterone and 18-hydroxycortisol in human adrenal glands. In the following paragraphs, insights, strengths, and limitations of GC-MS and LC-MS/MS in steroid profiling, as well as of IA in steroid measurements, will be specifically discussed and detailed in Figure 1 and in Table 1.

### 5.1. GC-MS in Steroid Analysis

The biological fluids most used in clinical practice for the determination of steroids in GC-MS are: serum [47,48,49], plasma [50], and urine [51,52].

The withdrawal of biological fluids, such as serum and plasma and therefore the measurements of the relative steroids, should be determined at a specific time during the day, considering the highly dynamic diurnal changes of steroid levels. The best time for taking the serum or plasma sample is in the morning, also considering outpatient practicality. However, the major disadvantage of this timepoint is that steroid levels are physiologically at their peak, making it difficult to distinguish between patients with adrenocortical hyperfunction from those with normal function. Therefore, 24-h urine collection may resolve this limitation, but it presents practical difficulties for patients. Thus, the collection of urine in the early hours of the morning could represent a good compromise [53]. Whatever the biological fluid of choice, the first step for quantification of steroid levels by GC-MS is to make the molecules more volatile and thermally stable: this is possible through derivatization procedures, often preceded by a purification step.

As described by Hansen et al., steroids from serum and plasma samples can be stabilized by adjusting the pH to 3.0 using sulfuric acid as a diluent. Subsequently, the solid phase extraction (SPE) and cleaning phases should be performed for the purification of the samples, and finally the derivatization phases are necessary to allow the evaporation of the steroid molecules before gas chromatographic separation [47].

Derivatization is one of the most important steps during sample preparation, broadening the range of detectable compounds, even in LC-MS/MS analysis, where derivatization is not a prerequisite; it is performed before the injection on the gas chromatograph to increase the molecules volatility and to enhance stability and signal intensity. Derivatization procedures are closely dependent on the functional groups of the analyzed molecules; in fact, in the case of steroids, the functional groups chosen for derivatization are oxo-groups and hydroxy groups linked to aliphatic and phenolic chains. Contrarily, steroids with sulfate groups should be hydrolyzed prior to analysis, without a direct derivatization phase [54]. Some steroids do not have a good chromatographic behavior, due to the presence of hydroxyl and ketone groups. Therefore, they are mainly derivatized by trimethylsilylation, using MSTFA, which binds to OH in position 17-b, with the help of some catalysts: (a) the addition of ammonium iodide, which allows the in situ formation of a trimethyliodosilane; (b) the addition of an imidazole base as a catalyst (TMSIM), used to derivatize only hydroxyl groups, by suppressing the enolization reactions. Furthermore, aldehydes or ketones are derivatized by oximation, exploiting their reactivity with primary amines. In the case of steroids, the oximation must be performed before the silylation of the remaining hydroxy groups, since this prevents the enolization of oxo-groups [54].

Despite the complexity of the sample preparation, GC-MS profiling has been widely used for steroid analysis, since it can provide simultaneous quantification of a panel of over 80 steroids [55].

The time required to obtain a result, considering not only the sample preparation time but also the runtime together with small batch sizes per run, is approximately 5–7 days, thus precluding the use of GC-MS for the screening of populations or for routine monitoring purposes [44].

Therefore, the complexity of sample preparation, as described above, is one of the main reasons why GC-MS methods, despite their usefulness for steroid profiling, are not suitable for the routine laboratory environment (Table 1). As a result, methods using MS coupled with liquid chromatography have become cutting edge for routine laboratories [53].

### 5.2. LC-MS/MS in Steroid Analysis

Currently, high performance LC (HPLC) is the analytical method of choice for measuring steroids, both because immunological tests suffer from wide cross-reactivity and because chromatography allows simultaneous measurement of hormones [56]. With the advent of technological innovations that have made possible to implement the robustness of the instruments and made them more accessible from an economic point of view, LC-MS/MS has been quickly incorporated into endocrine laboratories. In addition to the possibility of creating and validating “home-made” methods, it is now possible to make use, especially for the clinical practice, of ad hoc kits; considering that, a very detailed review describing the advantages and disadvantages of using home-made methods or ready-to-use kits for the determination of steroids in LC-MS/MS has been recently published by Le Goff et al. [57].

In short, the development of an internal method is not easy to achieve and requires the presence of highly specialized personnel. Instead, the use of ready-to-use kits allows a faster installation even for non-MS specialists (Table 1). On the other hand, an internal method is easier to manage as the technical information is all fully known by the operators, while in the case of ready-to-use kits information on the chromatographic column, mobile phase and sample preparation are normally unknown [57].

As already discussed in the previous section, the human matrices used for the quantification of hormones in LC-MS/MS are blood, serum [58], plasma, urine, saliva, and also tears [59]. In vitro models, for the measurement of the steroid profile in LC-MS/MS, have also been developed for the study of cell lines or tissues, such as adrenal glands, gonads and brain, with the aim of supporting mechanistic and functional investigations to obtain a profound knowledge about the perturbation of steroid metabolism [60].

Derivatization phase during sample preparation, which is the most important, delicate, and time-consuming step in the case of GC-MS, is not considered as a prerequisite in case of the LC-MS/MS analysis. Indeed, sample preparation is typically less complex than required for GC–MS.

Thus, the times required for obtaining a result are extremely reduced, keeping in mind the speed of sample preparation and also the times of runs in HPLC.

Furthermore, the ultra HPLC (UHPLC) instrumentation allows us to further shorten times, compared to traditional LC systems, making possible high productivity assays and allowing to obtain results in a few hours [44]. In any case, LC-MS/MS for the quantification of steroid hormones in clinical routine is still a technique that has some important disadvantages (Table 1). Firstly, analytes detection is subject to phenomena such as matrix effects and ion suppression, which could decrease the analytic performance. This is a crucial aspect in the correct management of LC-MS/MS analyses; in fact, if the ion suppression is not evaluated and corrected in an analytical method, the sensitivity of the method can be seriously compromised up to the impossibility of detecting the target analyte. This phenomenon is strictly linked to the variability of the sample matrix; for example, blood samples from different people can sometimes vary in some components of the matrix, and therefore show different ion suppression effects [61]. Secondly, isobaric interference may occur, which must be necessarily identified and controlled during the analytical validation phase, to avoid quantification errors. In the case of steroid profiling, isobaric interferences could lead to the detection and quantification of molecules that are structurally very similar from a chemical point of view, but functionally completely different, leading to possible erroneous clinical inferences.

Finally, it must be considered that the use of the LC-MS/MS techniques in steroid determinations is relatively young, and the reference intervals, essential for the interpretation of the results, are not yet well defined. On the other hand, reference intervals using immunofluorimetric techniques have been determined by over 50 years of studies and widely accepted; consequently, the reference ranges obtained from the new LC-MS/MS methods will still take some time to be generally accepted [57].

### 5.3. Imaging Approaches with MALDI Mass Spectrometry in Steroid Analysis

Imaging approaches with MALDI mass spectrometry (MALDI-IMS) is a versatile technique used to understand molecular mechanisms in biology and medicine, with particular application in the development and progression of cancer. This approach allows to detect and localize hundreds of endogenous biomolecules (metabolites, proteins, peptides, and lipids) or exogenous as drugs in intact tissue sections. However, the handling and preparation of the sample are crucial steps to obtain high quality and reproducibility of analysis. Typically, thin tissue sections are placed on a special slide, coated with a matrix that has the function of absorbing the energy coming from a laser source to extract the analytes of interest from the underlying tissue, which are then detected. The mass spectra acquired through the tissue are linked to defined geometric coordinates, which through specific algorithms allow to generate three-dimensional images of tissue sections [62].

Regarding the analysis of steroid profiles through MALDI-MSI, it must be said that the steroid synthesis pathways are complex as they have different structural isomers such as aldosterone and cortisone, which share the same chemical formula, but different biological functions. The techniques of choice for separating steroid isomers are chromatographic techniques, but these are not available in MSI. Currently, MS^n^ and separation of ion mobility coupled with MALDI or desorption electrospray ionization are potential techniques for discriminating between steroid isomers and determining their localization [63].

Notably, by using MALDI-MSI, it is also possible to visualize the tissue distribution of steroid hormones, a promising clinical application based on the study of the distribution of cortisol, Aldosterone and 18-hydroxycortisol in the human adrenal glands, as described by Takeo et al. [63].

The described method combines chemical processing, which includes the steps of derivatization and ionization of the analytes on the surface of the tissue, followed by the detection of their mass. Tissue chemical derivatization (OTCD) using the Girard’sT reagent allows for the detection and visualization of different steroid hormones by introducing a permanently cationic charged amine that reacts with the ketone group at the C3 position of the hormone A ring steroids [63].

The main feature of the MALDI-MSI approach is the possibility of correlating the distribution of steroid analytes with the anatomical composition of the tissue, bringing to light possible useful information in the follow-up phases (Table 1). Many efforts must be made to introduce this technique into clinical practice, bearing in mind that this approach is not applicable to biological fluids, but only to tissue samples and requires highly specialized personnel in sample manipulation.

## 6. Steroid Profiling as a Diagnostic Tool in Adrenocortical Carcinoma

About five decades ago, Gower et al. described alterations in steroid hormones production as possible markers of malignancy in adrenal tumors [64,65]. Subsequently, the application of steroid profiling in the diagnosis of adrenocortical disorders has been described on many occasions: firstly, by using difficult chromatographic separations and colorimetric or immunoassay procedures [66,67], more recently replaced by targeted metabolomics techniques using GC-MS and LC-MS/MS, respectively [4,68,69,70,71,72,73,74]. Even if not enough data on steroid profiling have been reported to be able to define a distinct malignant steroid fingerprint for ACC, biochemical evidence showed how most ACCs are characterized by accumulation of steroid precursor metabolites rather than by end products of steroidogenesis [75]. The most recent findings on steroid profiling by GC-MS and LC-MS/MS will be summarized to evaluate if steroid profiling might offer the key to solving the clinical dilemma in differentiating ACC from ACA. Moreover, such an approach would allow for a better assessment of the malignancy risk and the associated diagnosis, a treatment, and also the possibility of non-invasive follow-up for the patient.

Studies on the assessment of the diagnostic value of urinary steroid profiling by GC-MS revealed a specific fingerprint for ACC mostly coinciding with immature, early-stage steroidogenesis [6,75,76]. Arlt et al., in quantifying 32 distinct adrenal derived steroid by GC-MS in 24 h urine samples from 102 ACA patients and 45 ACC, identified a subset of nine steroids most informative in discriminating ACC from ACA. In particular, statistical comparison revealed that ACC patients show significantly higher excretion of metabolites derived from the precursors of androgen, mineralocorticoid, and glucocorticoid synthesis, without detecting any effect of age, sex, tumor size, or presence of metastasis on the over-described profile. Tetrahydro-11-deoxycortisol (THS), a metabolite of 11-deoxycortisol, was reported as the most discriminative steroid in differentiating ACC from ACA; the other eight were pregnenetriol (5-PT), pregnenediol (5-PD), pregnanetriol (PT), tetrahydro-11-deoxycorticosterone (THDOC), 5α-tetrahydro-11-dehydrocorticosterone (5αTHA), etiocholanolone (Etio), 5α-tetrahydrocortisol (5αTHF), and pregnanediol (PD) [6]. Following, Kerkhofs et al. aimed to determine the diagnostic performance of urinary steroid profiling in differentiating ACC from non-ACC in a large group of patients with adrenal tumors through the quantification of 22 selected steroids by GC-MS [76]. They found 18 steroid metabolites excreted in significantly higher concentrations in patients with ACC compared to patients with ACA or other adrenal conditions. Again, THS was demonstrated to have a very high sensitivity and specificity in differentiating between an ACC and a benign adrenal mass. Moreover, a significant correlation was found between THS excretion and ACC tumor size and stage. The confirmation of THS as the most discriminative marker of ACC in both studies suggests inhibition or lack of expression of 11-β-hydroxylase, which converts 11-deoxycortisol, a glucocorticoid precursor, to active cortisol [6,76]. Thus, it may be observed that urinary steroid profiling may offer an alternative diagnostic tool in the evaluation of malignancy risk of an adrenal tumor, especially if considering that an increased excretion of steroids in ACC, may occur without clinical signs of hormonal overproduction. Furthermore, the work conducted by Chortis et al. highlights the importance of using a steroid profile in ACC follow-up. In such a research initiative, urinary steroid metabolomics based on mass spectrometry combined with machine learning has been described as a novel diagnostic tool for the detection of postoperative relapses after microscopically complete (R0) resection of ACC, improving the ability to detect relapses of ACC [75]. Besides, it should be recognized that urinary steroid profiling by GC-MS exhibits several limitations making the transfer of this approach to clinical practice not easy [45].

Therefore, two more recent studies aimed to define steroid profiling by LC-MS/MS for diagnosing ACC in serum and plasma samples, respectively [4,7]. In fact, LC-MS/MS is the most promising analytical technique for measuring steroids in the clinical laboratory. Taylor et al. presented an LC-MS/MS method for the determination of 13 steroids, which they assessed in serum samples of 10 ACC and 38 non-ACC patients. The adrenal tumor groups they used for the study were age-matched with all samples collected in the morning in order to minimize the effects of age and diurnal variation [7]. In the comparison of LC-MS/MS steroid results between the ACC and the non-ACC groups a median of six steroids were found increased in the ACC group, with a high heterogeneity among patients. Importantly, 11-deoxycortisol was confirmed the most discriminating in differentiating ACC from non-ACC, further suggesting a defective 11-β-hydroxylase activity in ACC. As mentioned above, this enzyme catalyzes the conversion of 11-deoxycortisol to cortisol within the inner mitochondrial membrane. Thus, its activity may be particularly impaired in ACC. This result led them to speculate on the disruption of mitochondrial oxidative phosphorylation, common in cancer and well known with the term of “Warburg effect”. In 2019, Schweitzer et al. further evaluated the diagnostic potential of a 15-steroid plasma panel by LC-MS/MS analysis in 42 ACC and 66 ACA patients [4]. The following bioinformatic investigation showed significantly higher plasma concentrations of 11-deoxycorticosterone, progesterone, 17-OHP, 11-deoxycortisol, DHEA, DHEAS, and estradiol in ACC, compared to ACA patient samples. Furthermore, using the sex as additional information, they tried to optimize the model for male and female patients. Thus, they described two steroid signatures with only two hormones in common, androstenedione and DHEA, between male and female patients.

Intriguingly, both these studies on steroid profiling by LC-MS/MS revealed the increase of 11-deoxycortisol, 11-deoxycorticosterone, androstenedione and 17-OHP when comparing ACC with ACA patients, suggesting the clinical relevance of such steroids in [4,7].

More recently, Bancos et al. conducted a prospective multicenter evaluation of urine steroid metabolomics in the differential diagnosis of adrenocortical tumors study on 2017 adult participants with newly diagnosed adrenal masses, to validate the diagnostic accuracy of steroid metabolomic urinary test in LC-MS/MS also in combination with standard imaging protocols. The authors demonstrated high accuracy of urinary steroid metabolomics (AUROC 94.6%) in indicating a high risk of ACC, substantially improving the positive predicted value (PPV) compared to imaging tests. Additionally, the best performance was observed by combining tumor diameter, positive imaging characteristics, and urinary steroid metabolomics information. Interestingly, participants with a urinary steroid metabolomics result indicating a high risk of ACC could have undergone surgery for ACC earlier, and plus fewer unnecessary surgeries would have been performed [77].

## 7. Discussion

ACC is a rare malignant endocrine tumor. Although 60–70% of ACCs overproduce hormones, this is not clinically apparent in many cases. Since steroid production is relatively inefficient in ACC, hormonal markers could be a useful clue for the diagnosis and follow-up of ACC.

During the past decades, the use of LC-MS/MS has significantly increased in clinical chemistry laboratories, not only for research, but also for routine applications, thanks to its superior analytical specificity and to its ability to quantify a panel of analytes in a single measurement process [78,79].

For a long time, IA have represented the method of choice for steroid determinations, being rapid and easy to perform. However, the lack of specificity and matrix effects common to several steroid IA made its reliability questionable [80]. Nowadays, LC-MS/MS methods replaced IA for steroid quantifications, being able to provide higher sensitivity and specificity, and allow simultaneous determination of multiple steroids with rapid and simple sample preparation and acquisition (Figure 1) [43]. The complexity of LC-MS/MS analysis management may be overcome by the availability of ready-to-use diagnostic kits, which will likely facilitate access to this technology [4]. Although instrument prices and their maintenance may appear expensive, the possibility to analyze a large number of samples per day make the price of individual assays more affordable [81].

To date, steroid profiling by LC-MS/MS analysis has been evaluated and applied in many different steroid-related disorders. In fact, the diagnosis, prognosis, and management of such diseases strongly depend on accurate quantification of steroid hormones in biological fluids. Imbalances in production or metabolism of steroids lead to a wide range of steroid related disorders, sometimes complex adrenal diseases, including congenital adrenal hyperplasia (CAH) [71,72,73,74,82,83], adrenal cancer [4,6,7,45,75,76], hyperaldosteronism [45,84,85,86,87], Cushing’s syndrome [68], disorder of sexual development (DSD) [44], and polycystic ovary syndrome (PCOS) [44,69,88]. For some of these disorders, it is still open to debate on which steroids are most appropriate to be quantified for the related diagnosis. However, the great number of studies and clinical applications testify that multiplexed steroid profiling by MS technologies is increasingly playing a pivotal role in the diagnosis of these adrenal diseases, the panel or ratios of multiple steroid levels being much more informative and relevant than the value of any one alone. Moreover, LC-MS/MS methodology has the great advantage of being able to measure simultaneously steroids above and below the enzymatic block [89].

Indeed, as already described, alterations in the expression of steroidogenic enzymes in adrenal tumors often lead to the accumulation of steroid precursors and other steroid products that may immediately suggest at which level of steroidogenesis the defect lies [89].

However, it is important to point out that steroid hormones excess is not clinically evident in many of them [5]. To date, although tumor size alone is not sufficient, radiological examination is used for the evaluation of malignancy risk, as well as for the follow-up phases, generating psychological burden for patients. In this context, the use of LC-MS/MS steroid profiling could be a potential clinical tool for a rapid diagnosis and an early therapeutic treatment, ensuring a non-invasive follow-up for the patient.

## 8. Conclusions

In conclusion, LC-MS/MS serum steroid paneling offers a potentially important advancement in the clinical workup of patients with adrenal diseases by combining the measurement of both common and rarely measured steroids in a single analysis. Furthermore, LC-MS/MS steroid profiling could be the most informative test in the initial diagnostic approach of adrenal disorders and a valid support for the subsequent and expensive molecular diagnostic testing based on genetics, when required.

Interestingly, the over-discussed clinical studies for the detection of steroid profiling for ACC diagnosis revealed concordant results, even if further investigations and a complete validation in a larger cohort of patients is needed to better define the metabolic fingerprint required for the discrimination between ACC and ACA, as well as for the evaluation of malignancy risk.

Nowadays, it is well known that metabolites, in this specific case steroid hormones, represent the link between genotype and phenotype, and that the study of the metabolome, as well as of the steroidome, represents a significant advantage in detecting the end-point markers of biological events [90]. A metabolic fingerprinting of adrenal lesions, therefore, may provide clinically relevant information for diagnosis and treatment of adrenal disorders [35].

## Figures and Tables

**Table 1:** Advantages and disadvantages of Immunoassays (IA), Gas Chromatography-Mass Spectrometry (GC-MS) and Liquid Chromatography-Tandem Mass Spectrometry (LC-MS/MS) methods for the determination of steroids.

*Methods*	Advantages	Disadvantages
***IA***	very accessible and economic technique thanks to the large availability of ready-to-use kits for “traditional” steroids (cortisol, estradiol and testosterone)	the development of the less frequently used tests in routine clinical practice, could become laborious and expensive
	IA steroids quantification requires small quantities of samples, and very simple steps of sample preparation, thanks to the availability of reagents and low-cost instrumentation	only one analyte can be measured per immunoassay
	IA is considered an accessible technique, even to unskilled technicians	lack of specificity
	reference ranges of some steroids are fully accepted by the clinician community	the quantification of the steroids in IA is limited to the linear portion of the standard curve
		cross-reactivity between similar steroids
***GC-MS***	multiplexing analysis	time consuming analysis
	high specificity and sensibility	complexity of sample preparation; derivatization step is a prerequisite
	high resolution chromatographic separation, especially for steroids with similar chemical structures	cost effective analysis
		analysis not suitable for the routine laboratory
***LC-MS/MS***	analytical method of choice for steroids quantification	the development of an internal method is not easy to achieve and requires the presence of highly specialized personnel
	the use of ready-to-use kits allows a faster installation even for non-MS specialists	analytes detection is subject to matrix effects and ion suppression, decreasing analytic performance
	derivatization phase during sample preparation is not considered as a prerequisite	isobaric interference may occur
	reduced times for analysis	reference intervals, essential for the interpretation of the results, are not yet well defined
	high specificity and sensibility	
	multiplexing analysis	
***MALDI-MSI***	visualize the tissue distribution of steroid hormones	complex and crucial sample handling and preparation, applicable only to tissues
	Correlation of steroid distribution with anatomical composition of the tissue	Chromatographic techniques, which allow for the separation of steroid isomers, are not available in MSI.
